# High Sensitivity Design for Silicon-On-Insulator-Based Asymmetric Loop-Terminated Mach–Zehnder Interferometer

**DOI:** 10.3390/ma18040798

**Published:** 2025-02-11

**Authors:** Muhammad A. Butt

**Affiliations:** Institute of Microelectronics and Optoelectronics, Warsaw University of Technology, Koszykowa 75, 00-662 Warsaw, Poland; ali.butt@pw.edu.pl

**Keywords:** loop terminated Mach–Zehnder interferometer, silicon-on-insulator, Sagnac loop, subwavelength grating waveguide

## Abstract

This work presents a novel design for an asymmetric loop-terminated Mach–Zehnder interferometer (a-LT-MZI) based on a silicon-on-insulator (SOI) platform, tailored for refractive index (RI) sensing applications. A significant advantage of incorporating the Sagnac loop into the MZI configuration is its ability to reduce the interferometer’s effective length by half, offering a more compact design. This makes it ideal for integration into miniaturized optical devices, enabling space-efficient configurations without compromising precision or performance. The proposed device, featuring a pathlength difference (∆L) of 24.35 µm demonstrates a sensitivity of 261 nm/RIU, which is further enhanced to 510 nm/RIU by incorporating a subwavelength (SWG) waveguide in the asymmetric sensing arm. This modification boosts light–matter interaction, resulting in a larger shift in the interference fringes and significantly improving the sensor’s performance.

## 1. Introduction

The Mach–Zehnder interferometer (MZI) operates by splitting a coherent light beam into two paths using a beam splitter [1]. These paths are then recombined at a second beam splitter, producing interference based on the relative phase difference between the two paths [2,3]. This phase difference can be influenced by changes in the optical path length, caused by variations in temperature, pressure, the refractive index, or mechanical stress in the environment [4]. Due to its high sensitivity to these changes, the MZI is widely employed in sensing applications, such as chemical detection, strain measurement, temperature, and biosensing, by monitoring the interference pattern to detect subtle environmental variations [5,6,7].

Refractive index (RI) sensors play a crucial role in biosensing applications due to their high sensitivity and label-free detection capabilities [8]. These sensors operate by measuring changes in the refractive index at the sensor surface, which occur when biomolecules such as proteins, DNA, or antibodies bind to a functionalized sensing layer [9]. Techniques like surface plasmon resonance (SPR) and optical waveguide-based sensors utilize RI sensing to detect molecular interactions in real time with high precision [10,11]. RI sensors are widely used in medical diagnostics, environmental monitoring, and pharmaceutical research, enabling early disease detection, biomarker identification, and drug discovery [12]. Their ability to provide rapid, accurate, and non-invasive detection makes them an essential tool in modern biosensing technologies [13].

The Sagnac loop is a vital component in modern optical systems, particularly in the loop-terminated Mach–Zehnder interferometer (LT-MZI), where it significantly enhances both performance and functionality [14,15]. A key advantage of the Sagnac loop is its ability to reduce the interferometer’s effective length by half [16]. This compact design is highly advantageous for integration into miniaturized optical devices, enabling space-efficient configurations without compromising precision or performance. Furthermore, the loop enhances the interferometer’s phase sensitivity by increasing the effective optical path length through light recirculation. This amplification of interference effects permits the recognition of minute variations in the target parameters with exceptional accuracy. The Sagnac loop also offers superior common-mode rejection, a critical feature for eliminating noise and mitigating the impact of environmental disturbances [17]. By ensuring that common perturbations affecting both arms of the interferometer do not influence the interference pattern, the Sagnac loop provides highly stable and reliable measurements [15]. These combined attributes make the Sagnac loop indispensable in advanced interferometric sensors, such as those used for high-precision RI measurements and other sophisticated sensing applications [18,19].

In this work, an innovative a-LT-MZI structure on a silicon-on-insulator (SOI) platform is suggested for RI sensing applications. To further amplify the device’s sensitivity, an advanced asymmetric subwavelength grating loop-terminated Mach–Zehnder interferometer (a-SWG-LT-MZI) configuration is introduced. In this enhanced configuration, the sensing arm, originally made of a ridge waveguide (WG), is replaced with an SWG structure, which substantially enhances light–matter interaction. This modification enables more effective interaction between the guided light and the surrounding medium, resulting in an almost twofold enhancement in sensitivity compared to the a-LT-MZI structure based on a ridge WG. This approach not only improves the device’s performance but also underscores its potential for high precision and miniaturized sensing applications.

## 2. Sensor Design and Numerical Model

**Figure 1:**
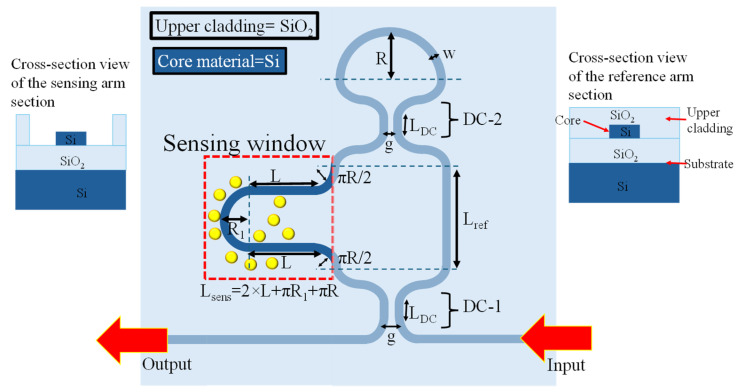
Two-dimensional scheme of an a-LT-MZI structure. The inset shows the cross-sectional view of the reference arm section (**right**) and sensing arm section (**left**). Yellow circles around the sensing arm represent analytes.

In this work, an a-LT-MZI structure on a silicon-on-insulator (SOI) platform is proposed for RI sensing applications. The schematic of the device is shown in Figure 1, and the geometric parameters of the device are elaborated in Table 1. The WGs are composed of silicon (Si), offering high RI contrast and excellent optical confinement. The device incorporates a silicon dioxide (SiO₂) upper cladding layer for enhanced protection and stability, with a strategically etched sensing window on the sensing arm. This window enables direct interaction between the guided optical mode and the surrounding environment, leading to a change in the effective refractive index (n_eff_) for efficient analyte detection. This change causes a shift in the interference pattern, which forms the basis for precise RI measurements.

The conditions for constructive and destructive interference in an a-LT-MZI used for refractive index sensing are determined by the phase difference between the two optical paths. Constructive interference occurs when the phase difference satisfies Δϕ = 2 mπ (m is an integer), resulting in maximum transmission, while destructive interference occurs when Δϕ = (2 m + 1)π, leading to minimal transmission. The loop reflects the waves back into the interferometer, and the output is extracted from the first DC (DC-1). As the RI of the medium around the sensing arm changes, it modifies the n_eff_ of the waveguide, thereby shifting the interference pattern. This makes the a-LT-MZI particularly effective for high-precision sensing applications.

The finite element method (FEM), implemented through COMSOL Multiphysics, provides a robust and precise computational approach for analyzing the transmission spectra and field distributions of a-LT-MZI structures. It enables the modeling of the complex geometry and material properties of the LT-MZI, accounting for variations in WG dimensions, refractive indices, and asymmetry. The material library in COMSOL provides a comprehensive database of material properties for accurate simulations. In this study, the refractive indices of Si and SiO_2_ were obtained directly from the COMSOL material library to ensure precision in optical modeling. By solving Maxwell’s equations in the spatial domain, FEM facilitates the computation of optical field distributions across the structure, offering detailed insights into mode coupling, interference effects, and localized field intensities. The transmission spectrum is analyzed over a wavelength range of 1520 nm to 1565 nm, providing a sufficiently broad span to capture multiple interference dips of the device with high precision. This range ensures a comprehensive assessment of the device’s spectral response and enables the detailed characterization of its interference behavior.

The proposed fabrication process for the a-LT-MZI structure consists of the following steps. First, the device layout can be designed to ensure precise structural and functional specifications. Next, electron beam (E-beam) lithography can be performed on a silicon-on-insulator (SOI) substrate to define the desired pattern with high resolution [20]. This can be followed by reactive ion etching (RIE) to transfer the pattern onto the substrate, shaping the device structure [21]. Subsequently, an upper cladding layer can be deposited to protect the device. Finally, a sensing window can be opened on the sensing arm using a controlled wet etching process. This proposed approach aims to achieve an accurate and well-defined fabrication of the photonic sensor.

## 3. Device Optimization

**Figure 2:**
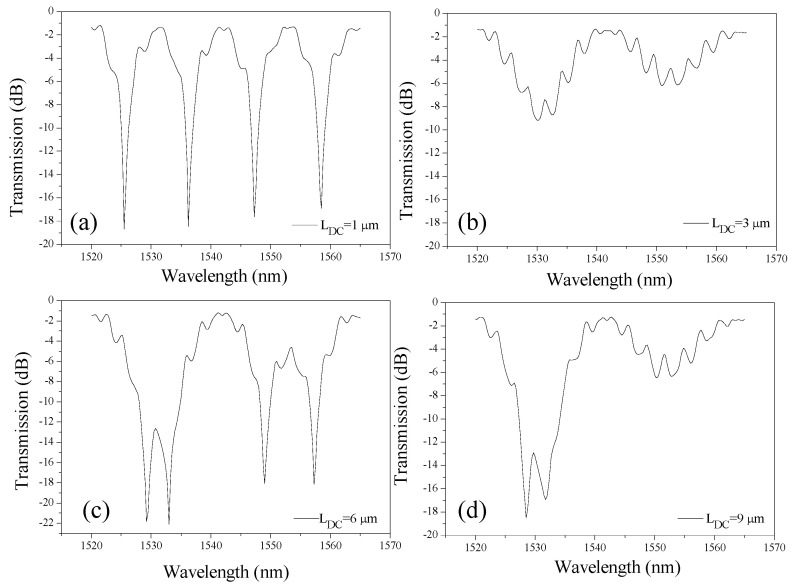
Transmission spectrum of an a-LT-MZI for different values of L_DC_: (**a**) 1 µm, (**b**) 3 µm, (**c**) 6 µm, and (**d**) 9 µm. The remaining geometric parameters of the device are kept constant: R = 5 µm, R_1_ = 2.5 µm, w = 400 nm, g = 150 nm, L_sens_ = 43.55 µm, and L_ref_ = 19.2 µm.

It is crucial to optimize the device geometry before utilizing it for sensing applications. In the first step, the length of the DC (L_DC_) is varied between 1 µm and 9 µm to identify the optimum length where distinct interference patterns are obtained. The optimal performance was observed for L_DC_ of 1 µm, where sharp and deep interference fringes were achieved (extinction ratio (ER) > 18 dB) (Figure 2a). This occurs because the power transfer is incomplete, resulting in an unbalanced splitting ratio that enhances the interference contrast. The feedback loop further amplifies the phase variations, reinforcing the intensity of constructive and destructive interference.

At 3 µm, which corresponds to the 3 dB coupling length, the interference dips become broader and shallower, likely due to a more balanced power splitting ratio, which reduces phase sensitivity and weakens feedback-induced modulation (Figure 2b). As L_DC_ increases to 6 µm and 9 µm, over-coupling effects emerge, where multiple power transfer cycles take place between the waveguides. While interference fringes remain sharp and deep, the FSR becomes irregular due to wavelength-dependent phase accumulation (Figure 2c). At L_DC_ = 9 µm, excessive coupling leads to overlapping interference dips, disrupting the periodicity of the interference pattern (Figure 2d).

The presence of an upper cladding affects the effective index and coupling coefficient of the DC. The cladding reduces the modal confinement in the silicon core, altering the coupling strength and phase evolution. This could contribute to the stronger performance at 1 µm, as the reduced coupling strength enhances phase sensitivity while still allowing effective interference. The combination of cladding-induced coupling effects, feedback phase accumulation, and power transfer characteristics suggests that shorter coupler lengths (~1 µm) provide the most optimized performance, while longer couplers (>3 µm) introduce over-coupling, leading to irregular FSR and dip overlapping.

**Figure 3:**
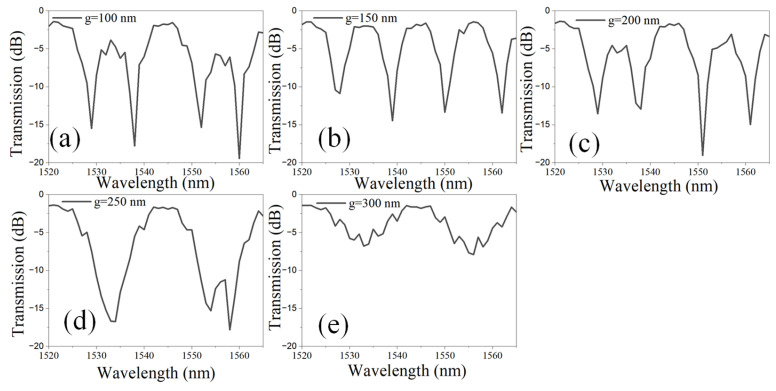
Transmission spectrum of an a-LT-MZI structure for different values of g: (**a**) 100 nm, (**b**) 150 nm, (**c**) 200 nm, (**d**) 250 nm, and (**e**) 300 nm. The remaining geometric parameters of the device are kept constant: R = 5 µm, R_1_ = 2.5 µm, w = 400 nm, L_DC_ = 1 µm, L_sens_ = 43.55 µm, and L_ref_ = 19.2 µm.

In the second step, the gap (g) between the WGs in the directional coupler (DC-1 and DC-2) with an L_DC_ of 1 µm is optimized to achieve optimal performance. In the DC, the separation g between two WGs plays a pivotal role in determining energy exchange through evanescent field interaction. The coupling coefficient (κ) exhibits an exponential dependence on g, given by κ ∝ e^−αg^, where α is a decay constant. A well-engineered gap ensures precise phase matching and minimizes insertion losses, crucial for maintaining signal integrity. In photonic integrated circuits (PICs), stringent control of g is essential for achieving the intended splitting ratio and optimizing overall device performance. The transmission spectrum of the LT-MZI structure is plotted for different values of g and ranges between 100 nm and 300 nm, as shown in Figure 3a–e. For g ranging from 100 nm to 200 nm, the interference dips exhibit a narrow linewidth (FWHM) and maintain a high ER exceeding 15 dB (Figure 3a–c). However, as g increases to 250 nm, the FWHM of the interference dips broadens, likely due to the overlap of adjacent dips, as illustrated in Figure 3d. When g reaches 300 nm, the interference dips become significantly wider, accompanied by a substantial reduction in the ER to approximately 7.9 dB, as shown in Figure 3e.

**Figure 4:**
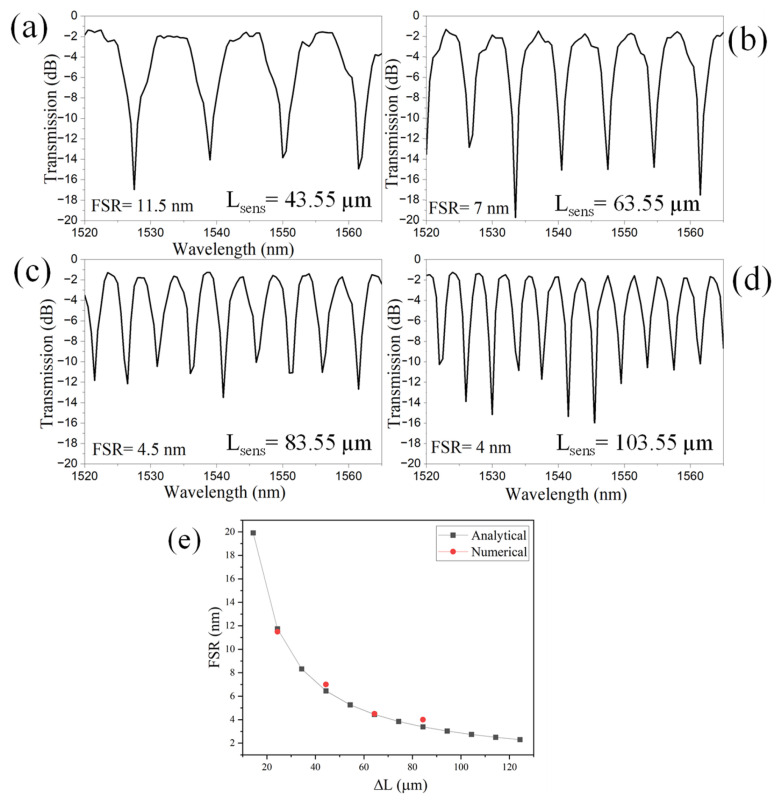
Transmission spectrum of an a-LT-MZI structure for varying L_sens_: (**a**) 10 µm, (**b**) 20 µm, (**c**) 30 µm, and (**d**) 40 µm. (**e**) FSR versus ∆L. The remaining geometric parameters are kept constant: R = 5 µm, R_1_ = 2.5 µm, w = 400 nm, L_DC_ = 1 µm, g = 150 nm, and L_ref_ = 19.2 µm.

The free spectral range (FSR) of an a-LT-MZI decreases as the path length difference (ΔL) between the sensing arm and the reference arm increases due to the inverse relationship between the FSR and the optical path length difference [22]. In the wavelength domain, the FSR is expressed as follows:

(1) $$FSR=\frac{\lambda^{2}}{2n_{g}∆L}\;$$

where λ is the operational wavelength, and n_g_ is the group index. Shamy et al. calculated the n_g_ of an SOI WG over a broad wavelength range between 1.5 µm and 1.6 µm, finding it to be approximately 4.2 [18]. Overall, as the path length difference increases, the phase difference between the two arms accumulates more rapidly, causing interference fringes to appear closer together in both frequency and wavelength domains, thereby decreasing the FSR [23]. The transmission spectrum of the LT-MZI structure is presented for a wavelength range of 1520 nm to 1565 nm, corresponding to the L_sens_ of 43.55 µm, 63.55 µm, 83.55 µm, and 103.55 µm, as depicted in Figure 4a–d. The FSR of the device decreases from 11.5 nm to 4 nm as ΔL increases from 24.35 µm to 84.35 µm. The analytical predictions derived from Equation (1) align closely with the numerical results, as illustrated in Figure 4e.

**Figure 5:**
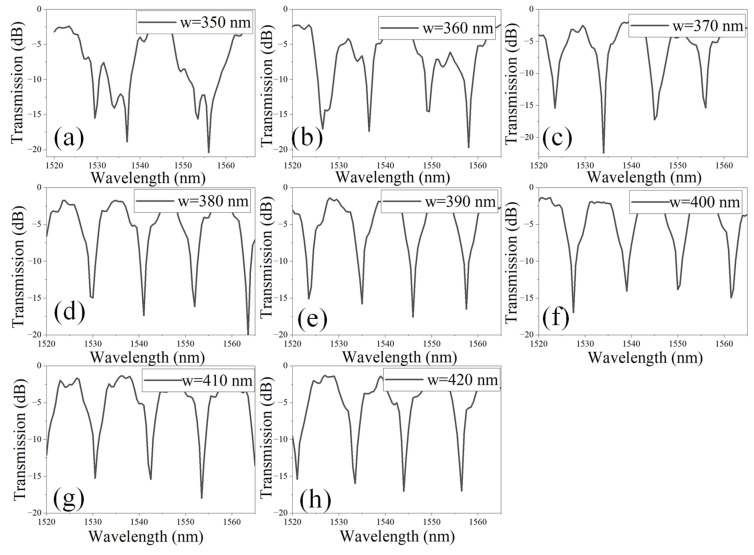
Transmission spectrum of an a-LT-MZI structure for different values of w: (**a**) 350 nm, (**b**) 360 nm, (**c**) 370 nm, (**d**) 380 nm, (**e**) 390 nm, (**f**) 400 nm, (**g**) 410 nm, and (**h**) 420 nm. The remaining geometric parameters are kept constant: R = 5 µm, R_1_ = 2.5 µm, g = 150 nm, L_DC_ = 1 µm, L_sens_ = 43.55 µm, and L_ref_ = 19.2 µm.

In the subsequent analysis, the impact of the WG width (w) on the interference pattern is evaluated over the wavelength range of 1520–1565 nm, as illustrated in Figure 5a–h. Notably, for w = 350 nm and 360 nm, significant distortion of the interference fringes is observed, accompanied by an increase in the FSR, as shown in Figure 4a,b. Such distorted interference patterns are undesirable for sensing applications, as they compromise the consistency and precision required for reliable measurements. The distortion of interference fringes and the widening of the FSR can be attributed to the higher-order dispersion effects, leading to phase mismatches between interfering modes and disrupting the coherence of the interference fringes. This emphasizes the need for optimizing WG dimensions to maintain well-defined and stable interference patterns suitable for high-precision sensing systems. For w ranging from 370 nm to 420 nm, the interference patterns are notably well defined, leading to the appearance of sharp and narrow dips in the transmission spectrum (Figure 5c–h). In optical interferometry, particularly in MZI devices, the FSR is primarily determined by the optical path length difference rather than the waveguide width. As a result, variations in width do not affect the FSR, which remains constant at 11 nm. This enhanced clarity and precision in the interference fringes indicates improved phase coherence and minimal distortion, making this range of WG widths highly suitable for applications requiring accurate and sensitive spectral measurements.

**Figure 6:**
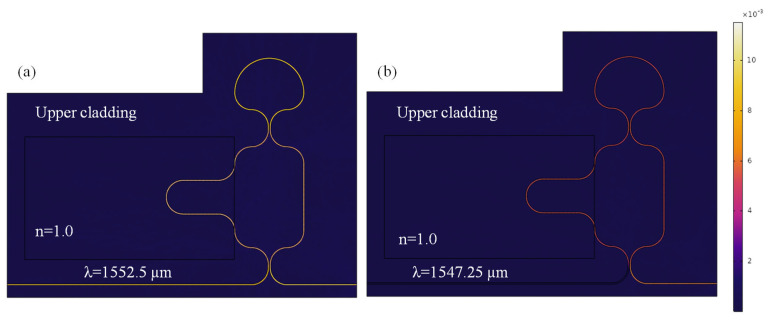
Normalized H-field distribution in the LT-MZI structure at an operational wavelength of (**a**) 1552.5 nm, and (**b**) 1547.25 nm. The geometric parameters of the device are as follows: R = 5 µm, R_1_ = 2.5 µm, w = 400 nm, g = 150 nm, L_DC_ = 1 µm, L_sens_ = 43.55 µm, and L_ref_ = 19.2 µm.

Figure 6a,b illustrate the normalized H-field distribution for the optimized LT-MZI structure, providing a detailed visualization of the interference mechanisms occurring at specific wavelengths. In particular, this figure highlights the phenomena of constructive interference at a wavelength of 1552.5 nm and destructive interference at 1547.25 nm. These interference effects arise due to the precise phase relationship between the optical waves propagating through the interferometer arms. In both cases, the RI of the medium surrounding the sensing arm is consistently maintained at 1.0, ensuring a controlled environment for evaluating the wavelength-dependent optical response of the structure. This analysis underscores the LT-MZI structure’s sensitivity to minute RI variations, making it highly effective for applications in optical sensing and biosensing.

## 4. RI Sensing Application

RI sensing using the MZI structures has emerged as a highly sensitive and versatile approach for detecting changes in the optical properties of a medium [24,25]. When the RI of the medium changes—due to the presence of a target substance or a biochemical interaction, the LT-MZI structure detects alterations in optical signals such as destructive interference wavelength [7]. The sensitivity of the device is calculated by utilizing Equation (2). This capability is particularly relevant for biosensing applications, where biological interactions like antibody–antigen binding, enzyme–substrate reactions, or nucleic acid hybridization can induce minute changes in the local refractive index.

(2) $$S=\frac{∆\lambda }{\Delta n\;}$$

where λ is the destructive interference wavelength, and n is the medium refractive index.

### 4.1. Design of a-SWG-LT-MZI

Subwavelength grating (SWG) WGs offer significant advantages over standard ridge WGs for sensing applications due to their enhanced light–matter interaction capabilities and increased design flexibility [26]. The periodic structure of SWG WGs allows for precise control of the effective refractive index, enabling the engineering of high-sensitivity photonic devices. Their ability to confine light in regions with high overlap between the optical field and the analyte enhances the interaction, improving the sensitivity of sensors [27]. Additionally, SWG WGs can support engineered dispersion and polarization control, which are critical in optimizing the performance of many sensing systems [28]. Compared to standard ridge WGs, they also facilitate the integration of advanced functionalities, such as slow-light effects and tailored modal profiles, further improving detection capabilities and enabling compact, highly efficient sensing platforms [28,29]. Recently, several highly sensitive photonic devices based on SWG WGs have been proposed for different sensing applications [27,30,31,32].

**Figure 7:**
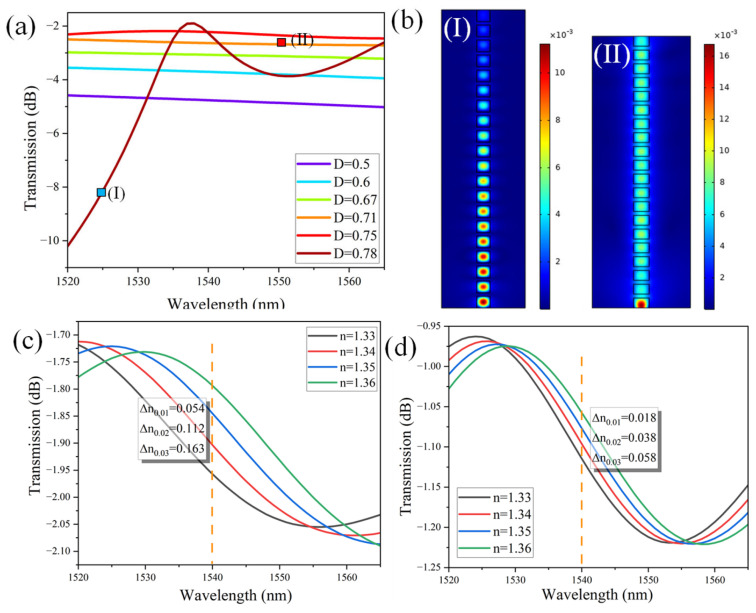
(**a**) Transmission spectrum of SWG WG versus wavelength spectrum in the range of 1520 nm and 1565 nm, (**b**) Norm. H-field distribution in the SWG WG, (**I**) for D = 0.78 and, (**II**) D = 0.71 at an operational wavelength of 1525 nm and 1550 nm, respectively. Transmission spectrum as a function of the ambient RI for (**c**) a SWG WG with D = 0.71 and (**d**) a standard ridge WG.

Initially, the geometry of the SWG WG is optimized to achieve optimal transmission, ensuring its seamless integration into the device for enhanced performance. The transmission spectrum of an SWG WG is plotted for different duty cycles (D) in the wavelength range of 1520 nm to 1565 nm, as shown in Figure 7a. D is calculated by utilizing Equation (3).

(3) $$D=\frac{width\;of\;grating\;element\;}{grating\;period}=\frac{a}{\Lambda }=\frac{a}{a+b}$$

where a and b are the length of the high index material (Si) and the length of the low index material (air), respectively. To suppress higher-order diffraction modes, Λ < λ/n_eff_. This ensures that only the fundamental mode propagates, enabling the WG to effectively behave as a homogeneous medium [33]. Within the subwavelength condition (D = 0.5 to 0.75), the structure suppresses Bragg reflections, allowing the SWG WG to operate similarly to a traditional WG with continuous material properties. However, when D approaches 0.78, Bragg reflections emerge in the transmission spectrum, obstructing the propagating mode and disrupting efficient WG operation. The normalized H-field distribution in the SWG WG is presented for D = 0.78 and D = 0.71 at operational wavelengths of 1525 nm and 1550 nm, respectively, as illustrated in Figure 7b. It is evident that, in case (I), the field fails to propagate to the end of the WG due to the presence of Bragg reflections, which disrupt the transmission. In contrast, in case (II), the field propagates seamlessly as a single Bloch mode, effectively emulating the behavior of a continuous mode in a homogeneous medium.

To validate the dominance of the SWG WG over the standard ridge WG, the sensitivity of both WG structures is estimated by monitoring the variation in transmission power as a function of changes in the RI of the surrounding medium (Figure 7c,d). This sensitivity is quantitatively assessed using the calculation provided in Equation (4). This comparison highlights the enhanced performance of the SWG WG in terms of its responsiveness to RI variations in the ambient medium.

(4) $$S_{waveguide}=\frac{Change\;in\;transmission\;power}{Change\;in\;refractive\;index\;of\;ambient\;medium}=\frac{∆T}{∆n};\;\;$$

The sensitivity of the SWG WG is approximately 5.4 dB/RIU, a remarkable improvement compared to the 1.9 dB/RIU sensitivity offered by a standard ridge WG. This substantial enhancement underscores the superior performance of the SWG WG in RI sensing. Furthermore, this analysis highlights that integrating the SWG WG into the a-LT-MZI structure will not only leverage its high sensitivity but also significantly elevate the overall detection efficiency of the system, making it a highly promising approach for advanced sensing applications.

**Figure 8:**
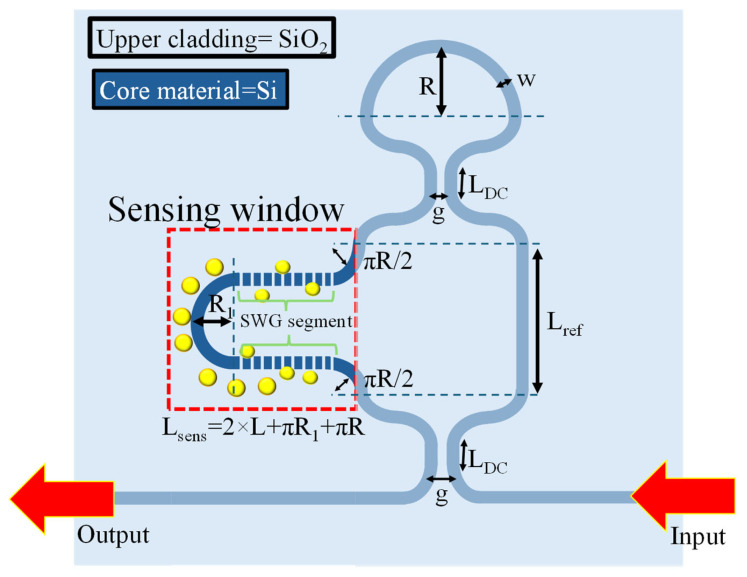
Two-dimensional scheme of a-SWG-LT-MZI structure. The straight segment of the sensing arm is replaced with SWG WG for better field overlap with the ambient medium. Yellow circles around the sensing arm represent analytes.

Building on the optimized geometry of the SWG WG, the straight segment of the sensing arm in the a-LT-MZI structure is replaced with a SWG WG featuring a duty cycle(D) = 0.67, as depicted in Figure 8. All other geometric parameters are maintained in alignment with the optimized values established in the preceding section, ensuring consistency in the design.

**Figure 9:**
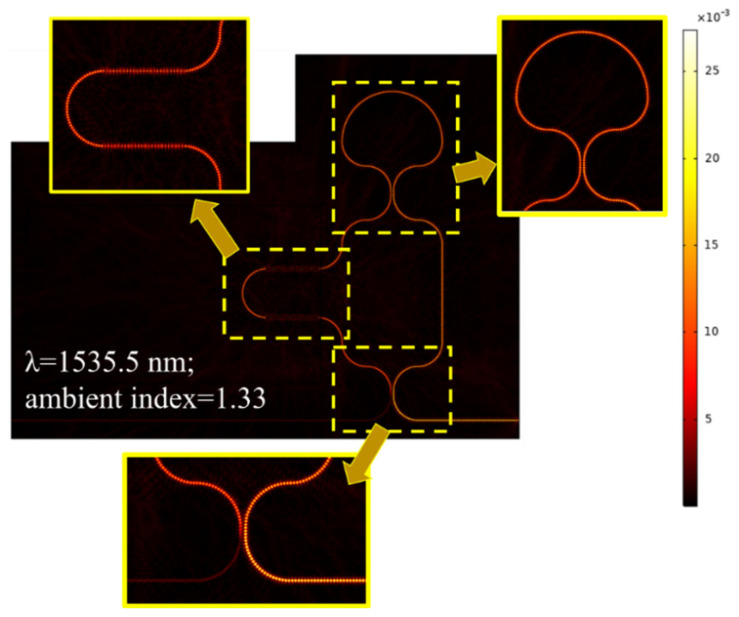
Normalized H-field distribution in the a-SWG-LT-MZI structure with ΔL = 24.35 µm at an operational wavelength of 1535.5 nm, with an ambient RI of 1.33 applied to the sensing arm. Inset the field distribution at different segments of the device.

Figure 9 presents the normalized H-field distribution in the a-LT-MZI structure at an operational wavelength of 1535.5 nm, with the sensing arm surrounded by a medium of RI = 1.33. The inset provides a detailed view of the field distribution in key regions: the loop segment (top-right), the SWG segment (top-left), and the DC. In DC, destructive interference occurs, effectively canceling the optical power and manifesting as an interference dip in the transmission spectrum. This detailed visualization highlights the critical role of each segment in the overall performance of the structure.

### 4.2. Sensing Performance of a-LT-MZI and SWG-a-LT-MZI Structures

**Figure 10:**
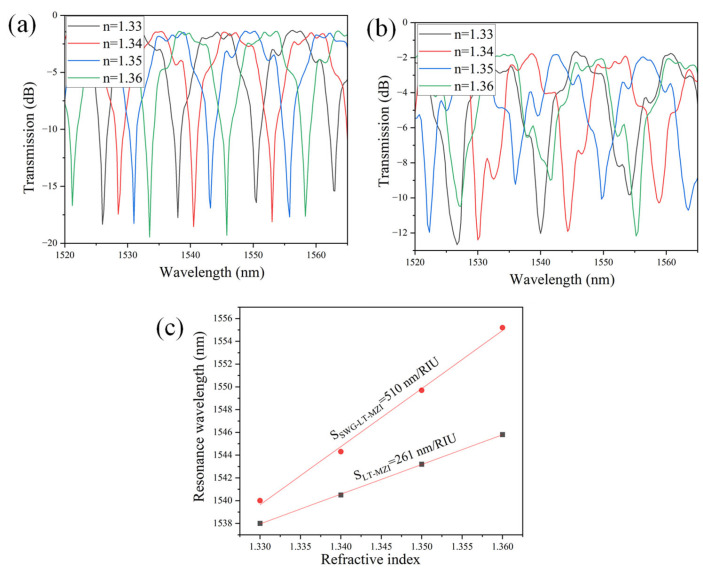
(**a**) Transmission spectra for the a-LT-MZI with ΔL = 24.35 µm under varying ambient refractive indices. (**b**) Transmission spectra for the SWG-a-LT-MZI with ΔL = 24.35 µm under the same conditions. (**c**) Comparison of the relationship between the resonance wavelength and the ambient RI for both configurations.

The sensing performance of the a-LT-MZI and SWG-a-LT-MZI structures having ΔL = 24.35 µm is analyzed by varying the RI of the medium covering their sensing arms. The transmission spectra, presented in Figure 10a,b for the a-LT-MZI and SWG-a-LT-MZI, respectively, demonstrate a pronounced redshift in the resonance wavelength as the RI increases from 1.33 to 1.36. This trend is further quantified in Figure 10c, which plots the resonance wavelength against the refractive index, highlighting a significantly steeper slope for the SWG-a-LT-MZI structure compared to the a-LT-MZI.

The sensitivity of the SWG-a-LT-MZI structure, defined as the ratio of the change in resonance wavelength to the change in the ambient refractive index (S = Δλ/Δn), is approximately 510 nm/RIU—nearly double that of the a-LT-MZI structure, which exhibits a sensitivity of around 261 nm/RIU. This marked improvement underscores the SWG-a-LT-MZI’s superior capability for detecting minute RI variations, making it highly suitable for advanced sensing applications. Table 2 provides a comparative analysis of the sensitivities of ring resonators and MZI structures implemented using different waveguide types, benchmarked against the findings of this study.

## 5. Conclusions

In this study, a high-sensitivity design for a silicon-on-insulator (SOI)-based asymmetric loop-terminated Mach–Zehnder Interferometer (a-LT-MZI) was presented and analyzed. By integrating a subwavelength grating (SWG) WG into the a-LT-MZI structure, significant enhancements in sensitivity were achieved compared to the a-LT-MZI. The SWG structure not only optimizes the effective RI modulation but also facilitates stronger interaction between the guided mode and the surrounding medium, resulting in a more pronounced phase shift for small RI changes. This analysis is conducted by employing the finite element method (FEM) via COMSOL Multiphysics software (version 5.5). The sensitivity of a-LT-MZI and SWG-a-LT-MZI with ∆L = 24.35 µm is estimated at 261 nm/RIU and 510 nm/RIU, respectively. This enhanced performance highlights the potential of the SWG-a-LT-MZI as a promising candidate for high-precision sensing applications, such as biochemical detection and environmental monitoring.

## Figures and Tables

**Table 1:** Geometric parameters of the device used in this study.

Parameters	Description	Values
R	The radius of the reflector	5 µm (fixed value)
R_1_	The radius of the loop of the sensing arm	2.5 µm (fixed)
w	Width of WG	350 nm to 420 nm
g	The gap between the WGs	100 nm to 300 nm
L_DC_	Length of directional coupler (DC-1 and DC-2)	1 µm to 9 µm
L_sens_	Length of sensing arm	2 × L + πR1 + πR. Where L = 10, 20, 30, 40 µm
L_sens_	Length of sensing arm for L = 10, 20, 30, 40 µm	43.55, 63.55, 83.55, 103.55 µm
L_ref_	Length of reference arm	19.2 µm
∆L	Differences in sensing arm and reference arm	24.35, 44.35, 64.35, 84.35 µm
λ	Operational wavelength region	1520 nm to 1565 nm

**Table 2:** Comparison of device performance with previous studies.

Design	Application	Sensitivity	References
Ring resonator	RI sensing	424 nm/RIU	[34]
Racetrack ring resonator	RI sensing	377.1 to 477.7 nm/RIU	[35]
Ring resonator	RI sensing	330 nm/RIU	[36]
MZI based on cascaded core-offset and microbending fiber structure	RI sensing	699.95 nm/RIU	[37]
Cladding etched PhC fiber MZI	RI sensing	211.53 to 359.37 nm/RIU	[38]
MZI based on waist-enlarged bitaper	RI sensing	287.65 nm/RIU	[39]
Hollow hybrid plasmonic MZI	RI sensing	160 nm/RIU	[36]
a-LT-MZI	RI sensing	261 nm/RIU	This work
SWG-a-LT-MZI	RI sensing	510 nm/RIU	This work

## Data Availability

The original contributions presented in this study are included in the article. Further inquiries can be directed to the corresponding author.
